# Epidemic Influenza: Notes on Its Origin and Method of Spread

**Published:** 1892-03

**Authors:** 


*Epidemic Influenza: Notes on its Origin and Method of Spread.* By Richard Sisley, M.D. Pp. xi., 150. London: Longmans, Green & Co. 1891.

*A Study of Influenza and the Laws of England concerning Infectious Diseases.* By Richard Sisley, M.D. [Second Edition]. Pp. 119. London: Longmans, Green & Co. 1892.

The first of these volumes was published in 1891, before the issue of Dr. Parsons' Report to the Local Government Board, and consists of notes confessedly incomplete, but compiled with considerable care, tending to show the manner of origin and spread of influenza; and, in particular, insisting upon the infectious nature of the malady and the consequent necessity for precautions against its spread, the neglect of which has, Dr. Sisley states, led to much suffering and to the loss of many lives.

The series of instances adduced by the author to prove the infectious nature of the disease has been abundantly confirmed by the results of Dr. Parsons' enquiry; but his further contention that notification of influenza should be compulsory under the Act of 1889, cannot be supported as likely to result in any useful control of its spread, for reasons fully set forth in the recent Provisional Memorandum of the Local Government Board of date January 23rd, 1892; chief amongst which are the indefinite and obscure symptoms of very many cases, and the shortness of the incubation period, leading to rapid and uncontrollable spread.

In the second volume under notice Dr. Sisley adopts a position with which we can cordially agree. "I am still of opinion," he writes, "that had a short Act of Parliament placed influenza amongst the diseases for which notification is compulsory, much sickness and many deaths would have been avoided. The good would have been done chiefly in an indirect manner. A discussion on such a Bill in Parliament would have concentrated public attention on the subject, the evidence of the Local Government Board would have received immediate and wide-spread recognition, and in this way people would have been taught that influenza was chiefly, if not entirely, spread by contagion, and they might have acted on their knowledge of that fact."

Dr. Sisley also points out that—apart from the application of legal enactments—individuals, families, and small communities may do much to avoid infection, and speaks in high terms of the action of the Dover authorities in warning persons, by their now historical document, of the danger of exposing themselves in public when suffering from influenza, and of the penalties attaching to the exposure of persons suffering from dangerous infectious disorders. In this commendation we heartily concur.

The volume contains, in addition, a summary of the laws in force relating to infectious disease, and the Provisional Memorandum on Influenza above referred to; and should prove useful to all who are concerned in public health administration.

